# Diseases of the Skin

**Published:** 1879-10-20

**Authors:** L. Duncan Bulkley

**Affiliations:** Physician to Skin Department, Demilt Dispensary, New York


### *LOCAL TREATMENT OF DISEASES OF THE SKIN.*

BY L. DUNCAN BULKLEY; A.M., M.D.,
Physician to Skin Department, Demilt Dispensary, New York.

SCABIES.—If there is any one disease which more than another demonstrates the advances in dermatology during the present century, that disease is scabies, and this more than any other establishes the value of a correct diagnosis and properly devised and effectually administered local treatment. In any large collection of books and monographs on skin diseases many essays can be found discussing the subject of the *internal* origin of "the itch," and the danger of curing it by local measures, which, of course, are entirely refuted by the practice of to-day; and as an extreme offset to this may be placed the fact that in the Hopital St.-Louis, Paris, these cases are never admitted to the hospital, but are cured by local treatment in less than two hours, the patient then returning to his home and occupation. As a basis or model the method there employed may be briefly stated.

The patient receives a ticket which passes him to the bath-house, and prescribes the course for the itch. The clothes are all taken off, wrapped in a bundle, and while the patient is undergoing the process they are submitted to a high dry temperature in an oven, heated, I believe, by superheated steam. The patient is now rubbed from head to foot with green soap (sapo viridis, a strong potash soft soap) for half an hour; then he enters a warm bath, remaining in it for another half-hour, and the third half-hour is occupied by a thorough and vigorous friction of the entire body with the following ointment:

℞ Sulphuris loti.......................................... ʒ ij.

Potass. carbonat......................................... ʒ iv.

Ung. simpl............................................... ℥ iij. M

This ointment is left on the body until the next day, to insure thorough action upon any acari which may be left, and to disinfect the clothes. The patient then, if desired, takes a bath on the following day, puts on clean clothes, and is cured, if the processes have each been conducted with sufficient diligence and vigor.

It may be somewhat difficult to carry out just this plan outside of a hospital, but with few modifications it is the one I resort to, both in public and private practice, and I tell the patients that they should be cured in a very short time if they will do their part.

Many skins in this country would be irritated to a very considerable degree by severe friction with a very strong sulphur ointment (and it is to be remembered that the pharmacopœial ointment is of the strength of one part of sulphur to two of lard), and various other measures are recommended and may be employed with good results. Especially is it necessary to use milder preparations with women and children, and perhaps the most common with me is the following:

℞ Styracis liquid.................................... ʒ ij to ʒ iv.

Unguent. sulphuris................................. ʒ ij to ʒ iv.

Unguent. aquæ rosæ, ad............................ ℥ ij.

M. Fiat unguent.

In strong men with tough skins I not unfrequently use the ordinary sulphur ointment in full strength.

The following is Hebra's ointment for scabies:

℞ Sulphuris flor., olei cadini............................aa ℥ i.

Saponis viridis, adipis.....................................aa ℥ ij.

Cretæ preparat............................................. ʒ vi.

M. Fiat unguent.

This is rather a strong application, and should not be used too vigorously on delicate skins.

One of the most difficult questions to decide often is how much of an eruption is due to the scabies and how much to the remedies employed; for not infrequently the eruption will seem to be much aggravated and it will be thought that the disease is on the increase, whereas renewed and augmented vigor of treatment will but still aggravate the case. In such cases it must be remembered that scabies *cannot* grow much worse rapidly, and if there has really been an active application of a well-known and well-tried remedy we may be pretty sure that the disease itself is arrested, that is, that the insects are killed, and we may safely wait, and by means of cooling remedies, internal and external, such as alkaline baths, etc., as before recommended for acute skin irritation, seek to allay the artificial eruption we have excited.

But again, it may then be necessary to give another course of the anti-parasitic remedies, if there still remains any itching after the subsidence of the acute symptoms; for we must always bear in mind the possibility that the applications were not thoroughly made, as directed, or also the possibility of reinfection from some other member of the family or from the clothes.

Scabies is not at all a frequent disease in this country, but cases of it occasionally occur which are a great opprobrium to the medical attendant from want of thoroughness of the cure. Some public institutions are hardly ever free from cases of scabies.

SCLERODERMA.—Little need be said of the local treatment of scleroderma, because, unfortunately, but little can be done internally or externally to arrest the progress of this distressing affection. The only measure which has been at all favorably reported on is galvanism, and that applied locally. I have had opportunity to follow this method satisfactorily in but a single case, which, however, yielded reasonably good results: the parts softened, and the limb and foot, which were before almost useless from the hide-bound condition of the skin, were restored to activity; when last seen, some years since, there was still, however, some of the stiffness remaining.

The mode of application consists in placing the positive pole upon a neighboring portion of healthy skin, while the negative electrode (a wet sponge or carbon) is passed over the affected part for a number of minutes, say averaging a quarter of an hour each sitting. The strength of the current is to be determined by the sensations of the patient; anywhere from five to twenty cells may be used.

Baths and emollient applications as ointments afford a certain amount of relief; my inclination would now be to employ the compound iodine ointment, used with patient friction, care being taken not to break the skin.

SCROFULODERMA.—Scrofuloderma, as distinct from ordinary lupus vulgaris, yields sometimes surprisingly to local treatment. Iodine takes first rank, and applications of the compound iodine ointment will occasionally give excellent results. Where greasy applications are not well borne, the compound solution of iodine will be found to be very serviceable, painted on the part or gently rubbed in.

Destructive remedies, caustics, etc., are not well borne by these indolent neoplastic growths, which either refuse to heal after their use or are followed by disfiguring cicatrices, which in turn take on the same phase of lowered vital growth.

SEBORRHŒA.—Local treatment must not be too much trusted to in any of the forms of seborrhœa, although the effect of local treatment is often very surprising. The subject is a very large one, and will have to be discussed in reference to the various forms in which this derangement of the sebaceous secretion occurs.

Commencing, then, for convenience, with the hairy scalp, we will remember that there are two forms of the affection seen here. First, that which best merits the name, that is, seborrhœa oleosa, but which is comparatively rare, where there is a real increase flux of oily matter, which sometimes, as we have seen it, occurs to the extent of making the scalp to be almost dripping with oil; the hair is disagreeable to handle and emits an unpleasant odor.

Here astringents are called for. The local treatment consists in part in frequently shampooing the scalp, and the most convenient application for this is a solution of the sapo viridis after the following formula :

℞ Saponis viridis .................................... ℥ iv.
Alcohol .................................... ℥ ij.
M. Filtra et adde :
Sprts. lavanduli .................................... ʒ j.
M. Faciat lotio.

Pouring a little on the head it is rubbed well with the fingers, a little water being sprinkled also on the scalp; this forms a good lather, which, after some further rubbing, may be washed off. Immediately after drying the hair, an ointment of tannin (ʒj ad ℥j) is to be well rubbed in to the roots of the hair, and to be reapplied every night. The washing is to be repeated every second or third night, or when the oiliness becomes obnoxious.

After using the ointment for a little while, say a week or so, a wash will probably serve better, and the following may be substituted for the ointment :

℞ Acidi carbolici .................................... ʒ ss.

Glycerini .................................... ʒ j.

Aquæ rosæ .................................... ℥ iv.

M. Faciat lotio.

Or we may at once proceed with a more stimulating application, and the following will serve a good purpose :

℞ Tinc. cantharid .................................... ʒ ij.

Tinc. capsici .................................... ʒ iv.

Olei ricini .................................... ʒ iij.

Sprts. vini rectif .................................... ℥ ij.

Sprts. roris marinæ, ad .................................... ℥ iv

M. Faciat lotio.

Care must be exercised in using this latter, as, in a skin with much tendency to eczema, it is capable of exciting considerable inflammation, as I have witnessed.

The more common form of seborrhœa of the scalp is seen in the form of more or less oily scales, which may accumulate into considerable masses, or may, becoming dry, fall continually and form much of the dandruff, often so very annoying. The local treatment of this, which is also an abnormal action of the sebaceous glands, is stimulating, like that just mentioned. If the scales are very thick and at all hard, so that washing would not remove them, the head may be first thoroughly soaked over night with some kind of oil—and the sweet almond oil recommends itself on account of its freedom from objectionable features, as rancidity, odor, etc. The next morning it is well washed off.

The washing may be accomplished by means of the alcoholic solution of soap previously mentioned, or tar soap answers very well, and is indeed a very common prescription with me.

After the washing it is even more imperative to have the subsequent application used immediately, that is, as soon as the scalp is at all dry, in order that the remedy may reach the affected part before there is an opportunity for the scales to reform. In my experience about the best application is citrine ointment diluted with three times the amount of the unguentum aquæ rosæ of the pharmacopœia, to which a few drops of an essential oil, as bergamot, geranium, or rose, may be added. Later on a small amount of cantharides may be added to the ointment with advantage.

This ointment is to be well and thoroughly rubbed into the roots of the hair; it is *not* to be put on the palm of the hand and applied as a pomade, but to be inserted with the ends of the fingers, generally by another party, deep among the hairs upon the surface of the scalp itself. The ointment is to be thus rubbed in daily, and the head may be washed as frequently as the greasiness of the scalp demands, generally twice or three times a week.

But I have seen cases where I believe that the disease was kept up by too frequent washings, and I give a caution to that effect.

The stimulating washes previously given are equally applicable in this dry form of seborrhœa capitis, and may be used with advantage. Sometimes ammonia suits the scalp better, and the following prescription will be found of great service :

℞ Tinc. capsici.......................................... ʒ iv.

Liq. ammoniæ.......................................... ʒ ij to ʒ iv.

Glycerin.......................................... ʒ ij to ʒ vi.

Aquæ rosæ, ad.......................................... ℥ iv.

M. Faciat lotio.

When the scalp is tender, and when the scales continue to form after this treatment, the following mild and very soothing wash, if continued for some time, will prove ultimately successful :

℞ Plumbi acetat.......................................... gr. viij.

Olei ricini.......................................... ʒ iv.

Olei bergam.......................................... ʒ ss to ʒ j.

Spts. vini rectif.......................................... ℥ iijss.

M. Faciat lotio.

But it must never be forgotten that seborrhœa is a local manifestation of general weakness, and that constitutional remedies are necessary to a cure.—*Arch. Dermatology.*

---

A MAP of Japan recently compiled at Yokohama from native sources shows that the Japanese are almost as strictly accurate in coast surveying as the best European naval officers.

